# Effect of picroside II on apoptosis induced by renal ischemia/reperfusion injury in rats

**DOI:** 10.3892/etm.2015.2192

**Published:** 2015-01-20

**Authors:** LEI WANG, XIUHENG LIU, HUI CHEN, ZHIYUAN CHEN, XIAODONG WENG, TAO QIU, LIN LIU

**Affiliations:** Department of Urology, Renmin Hospital of Wuhan University, Wuhan, Hubei 430060, P.R. China

**Keywords:** picroside II, ischemia and reperfusion injury, apoptosis

## Abstract

Renal ischemia and reperfusion (I/R) injury, which commonly occurs in kidney transplantation, is the leading cause of acute kidney injury. Picroside II possesses a wide range of pharmacological effects, including anti-apoptosis effects. In the present study, the ability of picroside II to attenuate apoptosis in a rat model of renal I/R injury was investigated. Sprague-Dawley rats were subjected to 45 min of ischemia followed by 24 h of reperfusion. Prior to reperfusion, the rats were treated with picroside II or an equal volume of phosphate-buffered saline. It was observed that renal function was significantly improved by the treatment with picroside II. Morphological analysis indicated that picroside II markedly reduced tissue damage and the expression of cleaved caspase-3. Reverse transcription-quantitative polymerase chain reaction and western blotting revealed that the expression levels of Bax and poly(ADP-ribose) polymerase-1 (PARP-1) were upregulated in the I/R group, whereas those of Bcl-2 were downregulated. However, the treatment with picroside II inhibited these changes induced by renal I/R injury. In conclusion, picroside II has potent anti-apoptotic activity against renal I/R injury.

## Introduction

Renal ischemia and reperfusion (I/R) injury is one of the main causes of acute kidney injury (AKI) and often occurs in such surgeries as kidney transplantation, partial nephrectomy, renal artery angioplasty, accidental or iatrogenic trauma, hydronephrosis and elective urological operations (1,2). AKI is characterized by a rapid decline in renal function, resulting in a failure to maintain fluid, electrolyte and acid-base homeostasis in clinical practice (3). Over the past decades, the incidence of AKI in adults and children has been continuously increasing, and attention paid to it has been growing due to its high morbidity and mortality rates as well as poor prognosis (4).

Renal I/R injury is a pathological process involving oxidative stress, inflammation reaction, calcium ion overloading and apoptosis. Cell apoptosis has been considered one of the most serious consequences of renal I/R injury in previous studies and determines the outcome of renal damage (5,6). As apoptosis is extremely important in renal I/R injury, the ideal therapeutic approach is to target its processes.

*Picrorhiza scrophulariiflora* belongs to the plant family, Scrophulariaceae. The roots of this plant are of benefit to health and often used in traditional Chinese medicine to treat a number of conditions (7). Extracts of the roots contain various terpenoids and glycosides (8) and picroside II is one of the main active constituents of the extracts. Numerous published studies have shown that picroside II has a wide range of pharmacological effects, including neuroprotective, hepatoprotective, anti-apoptosis, anti-cholestatic, anti-inflammatory and immune-modulating activities (9–11). However, to the best of our knowledge, it has not been demonstrated whether picroside II can protect renal tissue against apoptosis induced by renal I/R injury. Therefore, the major purpose of this study was to determine whether picroside II is able to attenuate apoptosis following renal I/R injury and its possible mechanism.

## Materials and methods

### Animal model of I/R

The present study was approved by the Institutional Animal Care and Use Committee of Wuhan University (Wuhan, China). Adult healthy male Sprague-Dawley rats, specific pathogen-free grade, weight 220–250 g, were supplied by the Experimental Animals Center of the Medical College of Wuhan University (Wuhan, China). This project was approved by the committee of experimental animals of Wuhan University, and the procedures were carried out according to routine animal-care guidelines. All experimental procedures complied with the Guide for the Care and Use of Laboratory Animals (1996). Briefly, rats were anesthetized with pentobarbital (45 mg/kg) and placed on a homeothermic table in order to maintain the core body temperature at 37°C. A midline laparotomy was made and right nephrectomy was performed. Subsequently, the left kidney was subjected to 45 min of ischemia followed by 24 h of reperfusion. All animals were randomly separated into three groups: Sham-operated (sham), I/R and picroside II groups. Each group contained eight rats. In the I/R and picroside II groups, after the right kidneys were removed, the left kidney vessels were clamped for 45 min followed by 24 h reperfusion. Rats in the sham group were only subjected to resection of the right kidney. The interventions were performed as described below.

### Intervention study

Picroside II (CAS No: 39012-20-9, purity >98%, molecular formula C_23_H_28_O_13_) was purchased from Tianjin Kuiqing Medical Technology Co. Ltd. (Tianjin, China). It was diluted to form a 10 g/l solution with 1 mol/l phosphate-buffered saline (PBS). Picroside II (10 mg/kg) 250 μl was administered via the tail vein to the rats in the picroside II group with a micro-syringe as described in a previous study (10), at the end of the 45 min of ischemia and prior to reperfusion. The rats in the I/R and sham groups were simultaneously injected with 1 mol/l PBS 250 μl. All mice were sacrificed following the 24 h reperfusion period with an overdose of pentobarbital sodium (Sigma-Aldrich, St. Louis, MO, USA), and the left kidneys were removed for the following experiments and the blood samples were collected for the detection of blood urea nitrogen (BUN) and creatinine (Cr) levels.

### Preservation of kidneys

The left kidney was removed under fully maintained anesthesia. After removal, the kidney was fixed in 10% phosphate-buffered formalin or immediately frozen, and stored at −80°C for subsequent experiments.

### Serum assays

At 24 h after I/R injury in every group, 1-ml blood samples were taken and analyses were performed according to the instructions of the Creatinine Assay and Urea Assay kits (Nanjing Jiancheng Bioengineering Institute, Nanjing, China). The absorbance was measured using a spectrophotometer (UV-1700; Shimadzu Corporation, Tokyo, Japan) and then the concentrations of BUN and Cr were calculated.

### Histological examinations

After the kidney fixed in 10% phosphate-buffered formalin, it was embedded with paraffin and sectioned to 4-μm thickness. The sections were deparaffinized and hydrated gradually, and stained with hematoxylin and eosin (H&E) and terminal deoxynucleotidyl transferase-mediated deoxyuridine triphosphate-biotin nick end labeling (TUNEL) techniques, respectively. Morphologic assessments were observed by an experienced renal pathologist who was unaware of the treatments. An established grading scale of 0–4, outlined by Jablonski *et al* (12), was used for the histopathologic assessment of I/R-induced damage.

### Apoptosis detection by the TUNEL method

The cell apoptosis was detected with a TUNEL assay according to the manufacturer’s instructions (Roche, Basel, Switzerland). Five fields were randomly selected and the number of positive cells was counted. The percentage of positive cells served as the apoptosis index.

### Immunohistochemistry

The expression of cleaved caspase-3 was conducted by immunohistochemical staining. Briefly, 4-μm sections were deparaffinized, and endogenous peroxidase activity was blocked with 3% hydrogen peroxide at 37°C for 10 min. Then the sections were treated with 10% normal goat serum in Tris-buffered saline (TBS) for 30 min at 37°C. Subsequently, they were incubated overnight at 4°C with polyclonal rat anti-cleaved caspase-3 (1:1,000; cat. no. #9661, Cell Signaling Technology, Boston, MA, USA). After washing three times with PBS, these sections were incubated with the secondary antibody from the UltraVision™ Quanto Detection System HRP DAB (Thermo Fisher Scientific, Waltham, MA, USA) for 30 min at room temperature, followed by color reagent 3,3′-diaminobenzidine (DAB). In the negative control group, the experiments were routinely performed.

### Reverse transcription-polymerase chain reaction (RT-qPCR)

Total RNA was isolated from the rat tissue from each group using TRIzol reagent (Invitrogen Life Technologies, Carlsbad, CA, USA) and the RNA concentration was obtained using a spectrophotometer. Single-stranded cDNA was synthesized using a cDNA synthesis kit (Takara, Kyoto, Japan) according to the instructions provided by the manufacturer. qPCR was performed with the Applied Biosystems SYBR Green mix kit (Applied Biosystems, Foster City, CA, USA) using the SLAN-96s Real-Time PCR system (Shanghai Hongshi Medical Technology Co., Ltd., Shanghai, China). The reaction composition contained: 2 μl cDNA, 12.5 μl 2X SYBR Green mix, 1 μl forward primer and 1 μl reverse primer and 8.5 μl ddH_2_O in a final volume of 25 μl. The primers used were as follows: Bax forward, 5′-TGAACTGGACAACAACATGGAG-3′, and reverse, 5′-AGCAAAGTAGAAAAGGGCAACC-3′ (GenBank accession number NM_017059); Bcl-2 forward, 5′-TTTGATTTCTCCTGGCTGTCT-3′ and reverse, 5′-CTGATTTGACCATTTGCCTG-3′ (GenBank accession number NM_016993); PARP-1 forward 5′-TCTCCAATCGCTTCTACACCCT-3′ and reverse, 5′-TACTGCTGTCATCAGACCCACC-3′ (GenBank accession number NM_013063). β-actin was used as a housekeeping gene. The data are presented as a ratio of gene to β-actin mRNA [sense: 5′-TGCTATGTTGCCCTAGAC NM_017059 TTCG-3′ and antisense: 5′-GTTGGCATAGAGGTCTTTACGG-3′ (GenBank accession number NM_031144).

### Western blot analysis

Total proteins were extracted, and quantified using bicinchoninic acid method. Then, equivalent weights of protein (40 μg/lane) was separated on 10% SDS-PAGE gels and then transferred to a nitrocellulose membrane. The membranes were blocked with 5% non-fat milk in Tris-buffered saline and Tween 20 (TBST) buffer and then incubated with the following rabbit polyclonal primary antibodies: Bax (1:1,000 dilution; 2772s, Cell Signaling Technology, Boston, MA, USA), Bcl-2 (1:1,000 dilution; 2870s, Cell Signaling Technology) and poly(ADP-ribose) polymerase-1 (PARP-1) (1:1,000 dilution; sc-7150, Santa Cruz Biotechnology, Santa Cruz, CA, USA) at 4°C overnight. Subsequently, after being washed twice with PBS, the membranes were incubated with goat anti-rabbit horseradish peroxidase-conjugated immunoglobulin G secondary antibody (1:2,000; ZDR-5306, ZSGB-BIO, Beijing, China) at room temperature for 1 h. Specific bands were visualized using Immobilon Western Chemiluminescence HRP substrate (Merck Millipore, Darsmtadt, Germany). Optical densities were detected using Quantity One software (Bio-Rad, Hercules, CA, USA).

### Statistical analysis

Data are presented as the mean ± standard error of the mean. Statistical analyses were conducted using SPSS version 17.0 (SPSS Inc., Chicago, IL, USA). The means of the different groups were compared using one-way analysis of variance and Student-Newman-Keuls test. Differences were considered statistically significant when P<0.05.

## Results

### Renal function

**Figure 1:**
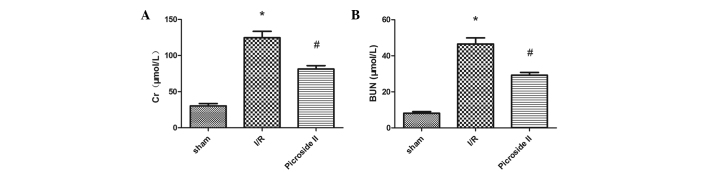
Effect of picroside II on (A) the serum Cr concentration and (B) the serum BUN concentration after 45 min of ischemia followed by 24 h of reperfusion. Bars represent the mean ± standard error of the mean (n=6). ^*^P<0.05 vs. the sham group; ^#^P<0.05 vs. the I/R group. Cr, creatinine; BUN, blood urea nitrogen; I/R, ischemia and reperfusion.

It was evident from the results that rats subjected to I/R injury showed significant increases in BUN and Cr levels compared with rats in the sham group. The renal function damage induced by I/R was significantly improved by the treatment with picroside II (P<0.05; Fig. 1).

### Histopathology

**Figure 2:**
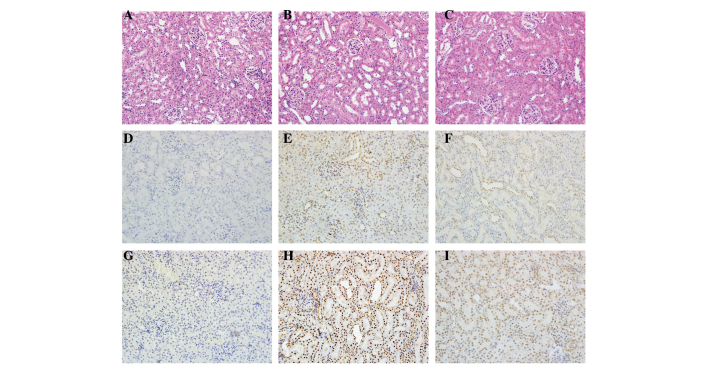
Histological features were evaluated by H&E and TUNEL staining and immunohistochemistry was performed to evaluate the expression of cleaved caspase-3. Representative kidney sections stained with (A-C) H&E, (D-F) TUNEL and (G-I) cleaved caspase-3 (brown nuclear staining) in kidneys at the end of the 24 h reperfusion period. Sections from (A,D,G) a sham-operated rat, (B,E,H) a rat subjected to I/R and (C,F,I) a rat subjected to picroside II treatment. All images of H&E, TUNEL and immunohistochemical staining, original magnification ×200. H&E, hematoxylin and eosin; TUNEL, terminal deoxynucleotidyl transferase-mediated deoxyuridine triphosphate-biotin nick end labeling; I/R, ischemia and reperfusion.

**Figure 3:**
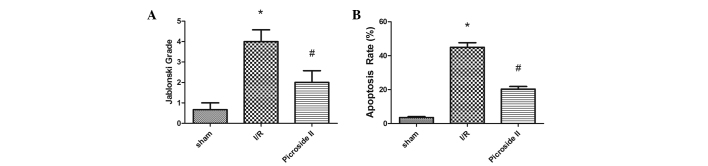
(A) Effect of picroside II on the Jablonski grading scale scores at 24 h after reperfusion. I/R induced severe lesions in the kidneys of rats and picroside II relieved these lesions. (B) Effect of picroside II on the apoptosis index at 24 h after reperfusion. Bars represent the mean ± standard error of the mean (n=6). ^*^P<0.05 vs. the sham group; ^#^P<0.05 vs. the I/R group. I/R, ischemia and reperfusion.

Renal I/R resulted in significant renal injury, as evidenced by tubular necrosis, medullary hemorrhage and congestion. However, treatment with picroside II reduced these types of severe renal damage (Fig. 2A–C). According to the Jablonski scores, 45 min of renal ischemia followed by 24 h of reperfusion resulted in severe acute tubular necrosis. Quantitative analysis showed an clearly decreased score in the picroside II group compared with the I/R group (Fig. 3A).

### TUNEL staining

At 24 h after I/R, no TUNEL-positive cells were present in the sham group. In the I/R group, positive cells were easily observed. However, there were clearly fewer TUNEL-positive cells in the picroside II group compared with the I/R group (Fig. 2D–F). The mean percentage of TUNEL positive cells in the I/R group was greater than that in the sham and picroside II groups (Fig. 3B).

### Immunohistochemistry

In this study, cleaved caspase-3 was detected by immunohistochemical techniques. Staining revealed that cleaved caspase-3 was rarely found in the kidneys of the rats in the sham group. However, in the I/R group, the rat renal tissues were strongly positive for cleaved caspase-3 expression. Compared with the I/R group, the expression of cleaved caspase-3 was reduced in the picroside II group (Fig. 2G–I).

### RT-qPCR analysis

**Figure 4:**
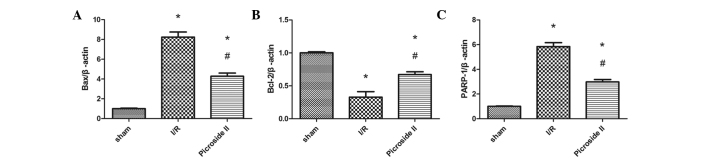
mRNA levels of Bax, Bcl-2 and PARP-1 in the kidney. Effect of picroside II on the mRNA level of (A) Bax, (B) Bcl-2 and (C) PARP-1 after 45 min of ischemia followed by 24 h of reperfusion. mRNA was standardized by β-actin mRNA. ^*^P<0.05 vs. the sham group; ^#^P<0.05 vs. the I/R group. PARP, poly(ADP-ribose) polymerase-1; I/R, ischemia and reperfusion.

The relative mRNA expression levels of Bax, Bcl-2 and PARP-1 to β-actin were determined. The mRNA levels of Bax and PARP-1 were significantly greater in the I/R group than in the sham group. However, the treatment with picroside II was found to significantly reduce the mRNA expression levels of Bax and PARP-1 following I/R (Fig. 4).

### Western blot analysis

**Figure 5:**
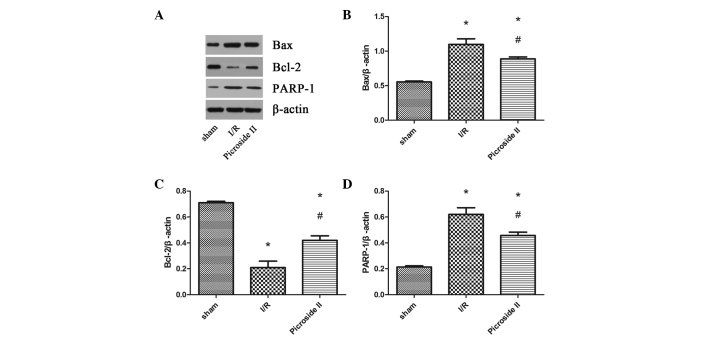
(A) Representative western blots showing the effects of picroside II on Bax, Bcl-2 and PARP-1 expression in the kidney after 45 min of ischemia followed by 24 h of reperfusion. β-actin was used to show equal amounts of protein loading in each lane. Relative band densities of (B) Bax, (C) Bcl-2 and (D) PARP-1 to the mean value of the control. ^*^P<0.05 vs. the sham group; ^#^P<0.05 vs. the I/R group. PARP, poly(ADP-ribose) polymerase-1; I/R, ischemia and reperfusion.

To investigate the different levels of protein expression, the expression of Bax, Bcl-2 and PARP-1 was analyzed by western blotting (Fig. 5). It was evident from the results that the expression levels of Bax and PARP-1 were upregulated in the I/R and picroside II groups compared with the levels in the sham group. However, picroside II attenuate these increases in expression induced by I/R. Bcl-2 levels were downregulated in rats subjected to I/R compared with those in the sham group, but in the picroside II group, the expression level was clearly greater than that observed in the I/R group.

## Discussion

AKI is a common clinical complication, with a rapid reduction in the glomerular filtration rate. Although there are numerous renal replacement therapies, the morbidity and mortality rates associated with AKI remain high (13). Currently, the main therapy is focused on nutritional and supportive care and there is no specific therapy that can be used to significantly improve the clinical outcome of patients with AKI (14). Therefore, it is necessary to seek new therapeutic strategies for AKI.

Picroside II is one of the main active constituents of the extracts of *Picrorhiza scrophulariiflora* Pennell and it has been shown to possess a wide range of pharmacological effects, including neuroprotective, hepatoprotective, anti-apoptosis, anti-cholestatic, anti-inflammatory and immune-modulating activities (15–17). In a previous study, it was demonstrated that picroside II can inhibit apoptosis in rats subjected to focal cerebral I/R injury (10). Another study reported that picroside II protects hepatocytes against injury and counteracts apoptosis by maintaining the integrity of the mitochondrial membrane and enhancing the activity of ATPase in mitochondria (11). However, to the best of our knowledge, it has not been demonstrated whether picroside II is able to protect renal tissue against renal I/R injury. In the present study, an investigation of whether picroside II could attenuate the apoptosis induced by renal I/R injury in rats was conducted.

It is generally accepted that apoptosis leads to renal dysfunction subsequent to ischemia (18–20). This is based on studies using various approaches (including caspase-3 activity, Bax activation, cytochrome *c* release and changes in the Bcl-2/Bax ratio) that have demonstrated that apoptosis is a consequence of ischemia (21). Apoptotic mechanisms are complex and the caspase enzyme cascade plays a key role among the multiple mediators of the complex process of apoptosis. Caspase-3 is an important enzyme among the cysteine proteases that exist as inactive zymogens. It is the most crucial downstream apoptosis protease in the caspase cascade ‘waterfall’ (22). Its activation is determined by a series of signal transduction cascades, among which the interaction between Bcl-2 and Bax plays a key role (23). The results of the present study indicated that picroside II significantly reduced apoptosis caused by I/R injury, which was demonstrated by TUNEL staining. Immunohistochemical staining revealed that the expression of cleaved caspase-3 was clearly greater in the I/R group than in the sham group, and that treatment with picroside II significantly reduced its expression, which was consistent with the results of TUNEL staining.

To clarify the protective mechanisms of picroside II, the expression levels of certain important apoptotic molecules were evaluated. The proteins of the Bcl-2 family, which are crucial regulatory factors, promote either cell survival (e.g., Bcl-2) or cell death by apoptosis (e.g., Bax). The ratio of Bcl-2/Bax is a pivotal factor that determines whether or not apoptosis occurs in cells exposed to injury (24). Also, activated caspase-3 can hydrolyze PARP, which is a type of catalytic protease. RT-qPCR and western blotting indicated that the expression levels of Bax and PARP-1 were upregulated and the expression level of Bcl-2 was downregulated in the I/R group, and that picroside II treatment was clearly able to ameliorate these changes in expression induced by I/R. In addition, the Bcl-2/Bax expression ratio decreased markedly in the I/R group compared with that in the sham group, but increased significantly with picroside II treatment, indicating that picroside II attenuated apoptosis by affecting the Bcl-2/Bax expression ratio.

In conclusion, the present study demonstrated for the first time that picroside II is able to protect renal tissue against I/R injury. This protective effect may be achieved through the inhibition of apoptosis. Therefore, these findings reveal the potential role of picroside II as a therapeutic option for AKI.

## Abbreviations

AKI: acute kidney injury
I/R: ischemia and reperfusion
